# The Care of Abandoned Children

**Published:** 1870-11

**Authors:** 


### THE CARE OF ABANDONED CHILDREN.

Prof. Jacobi, a high authority on all matters connected with the treatment of children, and one of the physicians of the Infants' Hospital on Randall's Island, and of the Nursery and Childs' Hospital in this city, has published an important report concerning the mortality of infants in institutions devoted to their aggregation and rearing, both here and abroad, with statistical tables which would rejoice the heart of Herod the Tetrarch, or relieve the Rev. Mr. Malthus's mind of all immediate apprehensions concerning the increase of population beyond the means of subsistence, were either of these worthies alive to profit by the information.

Without following all the numerical details embraced in this report, it may suffice to state, in general terms, that the death-rate among children under two years old, seems to be more than twice as great within the most carefully tended establishments where numbers of them are gathered together, as without the walls of these charities. Collating the mortuary records of eleven European countries, (and these dated some years ago, when the sanitary conditions were probably worse than now), the average percentage of deaths during the first year, even including still births, is 20.85, counting the total mortality in palace and hovel alike; while in foundling asylums and hospitals, these figures are commonly doubled, and in some instances more than tripled. Of "abandoned children" coming under official care, the deaths range from 50 to 100 per cent.

The estimates thus far relate to foreign countries; but on turning our glance homewards, the prospect affords no greater encouragement. In the Nursery and Child's Hospital, which is under the painstaking management of a number of most estimable ladies, and the Infant's Hospital, controlled by the Commissioners of Charities and Correction, the mortality, despite all care and watchfulness, is frightful. In the former, to which the children were admitted at the average age of 4½ months, the rate has been 40 deaths to 60 admissions, or even counting infants discharged after sixteen days' stay in the institution, 50 per cent. of all inmates under two years of age; and Prof. Jacobi adds:

"It appears that the mortality of the nursery, if all of the admitted infants were newborn instead of being 4–5 months, would be so appalling that I am glad I am not required to state its exact figures. The worst figures of the European foundling hells of former centuries are not more fearful than ours, and although being an officer of that institution myself, and believing that I and all the rest of us have conscientiously tried to do our duties, I cannot but testify and bow down to the truth, phat in spite of all the efforts of the medical staff, and the painstaking of kind-hearted and self-sacrificing ladies, the probability of the lives of children entrusted to a public institution is very slim indeed. The younger the children, and the larger the institution—the surer is death. Every story added to an edifice which is meant to be a temple of love, is an additional hecatomb of the innocents. Modern civilization, planning for the best, but mistaken about the means, has succeeded n out-Heroding Herod."

And again:

"Thus, the nursery, losing 36.5 children of 100 admitted at 4–5 months, would lose 73 of 100 admitted at birth, *viz.:* more than 2½ times as many as the average deaths of newborn children before the end of their first year in the whole city of New York."

As regards the Infants' Hospital, it is stated that the mortality rates "do not look any more promising; on the contrary, they are worse in proportion to the poorer condition in which the infants are received, and the numerous and well-known drawbacks our institution is suffering from." Notwithstanding that the average age of admission is four months and 14 days, thus excluding nearly two-thirds of the deaths usually occurring in the first year of infancy, the rate of decease in 1869 was 59.7 per cent., and even this is an improvement over former years.

Such figures as these bear sadly conclusive evidence as to the worse than uselessness of philanthrophic schemes which involve the aggregation of numerous inmates under the same roof; and yet the great question remains, what are we to do for the forlorn little waifs and strays of humanity that abound in all large cities? Means might, perhaps, be devised for improving the condition of infants cared for at home by even the poorest parents; but we should still have to provide for the multitude of illegitimate and abandoned children who are necessarily dependent on public charity for shelter. How this may be best accomplished is a matter of which the practical settlement lies in the future. One thing is clear; the crowding together of large numbers must be avoided, and separation practiced as far as possible. In France, the best results are obtained where children are distributed in country farm-houses for care; but there the system is under rigid governmental supervision, and the developments of "baby-farming" in England show the frightful dangers of such a plan when exempted from authoritative control. Under our republican institutions, it would be impracticable, even were it permissible, for the government to assume the direct management of affairs of this sort, and they must be left, as now, to private benevolence.

We believe that the solution of the problem will ultimately be found in the adoption of the "cottage system;" in the abanment of large asylums and hospitals, and the erection, in their stead, of numerous small and inexpensive structures—little sanitary villages, so to speak—in the pure air beyond the city limits. The Cottage-Hospital at Ventnor, England, has been found to work admirably in the case of sick adults, and we are convinced that a similar plan for the rearing of infants, under such careful management as that of the Nursery and Childs' Hospital, would incalculably reduce the death-rate among the little ones taken in charge.—*N. Y. Medical Gazette.*

---

Cancer of Uterus.—Professor Fordyce Barker, of New York, advocates in those cases where there is extreme sensitiveness of the ulcerated surface, pessaries of iodoform.

℞. Iodoform .............................. gr. x.

Butyri cacao .............................. ʒ j.

Glycerine .............................. gtt. v. M

One pessary. This also serves to correct fetor. A dracham of iodoform to the ounce of lard, is also a beneficial ointment. —*New York Medical Record.*

